# MT1-MMP in breast cancer: induction of VEGF-C correlates with metastasis and poor prognosis

**DOI:** 10.1186/1475-2867-13-98

**Published:** 2013-10-13

**Authors:** Guangyu Yao, Ping He, Lujia Chen, Xiaolei Hu, Fan Gu, Changsheng Ye

**Affiliations:** 1 Breast Center, Nanfang Hospital, Southern Medical University, Guangzhou 510515, Guangdong Province, China; 2 Department of Pathology, The First Affiliated Hospital of Guangzhou Medical University, Guangzhou 510120, Guangdong Province, China

**Keywords:** Membrane-type matrix 1 metalloproteinases, Vascular endothelial growth factor-C, Lymphangiogenesis, Metastasis, Breast cancer

## Abstract

**Background:**

Recent evidence suggests that vascular endothelial growth factor-C (VEGF-C)- dependent tumour production promotes lymphangiogenesis, while membrane-type matrix 1 metalloproteinase (MT1-MMP) is involved in the critical steps leading to carcinogenesis. However, the role of MT1-MMP in lymphangiogenesis and lymphatic metastasis remains poorly understood. In the present study, we investigated the relationship between MT1-MMP and VEGF-C in human breast cancer and correlated MT1-MMP and VEGF-C expression with lymphangiogenesis and prognosis.

**Methods:**

MT1-MMP and VEGF-C levels were compared in five breast carcinoma cell lines. We used a membrane invasion assay to assess the effect of MT1-MMP and VEGF-C expression, as well as anti-MT1-MMP and VEGF-C antibodies, on cancer cell invasion. We further assessed MT1-MMP and VEGF-C immunoreactivity and lymph vessels in a cohort of human breast cancer specimens (n = 106) and associated MT1-MMP and VEGF-C expression with clinicopathological parameters, such as lymphatic vessel density (LVD), and patient prognosis.

**Results:**

MT1-MMP and VEGF-C expression differed among the five breast cancer cell lines and MT1-MMP and VEGF-C expression were correlated with tumour cell invasion. VEGF-C mRNA expression levels and invasive activity of MDA-MB-231 cells was inhibited by an anti-MT1-MMP antibody in a concentration-dependent manner. A significant correlation was found between the expression of MT1-MMP and VEGF-C in breast cancer patient samples and elevated MT1-MMP and VEGF-C expression was associated with higher LVD, lymph node metastasis, cancer stage, and a decline in overall survival rates.

**Conclusions:**

Our data demonstrate that MT1-MMP expression is closely correlated with VEGF-C expression, and that MT1-MMP promotes lymphangiogenesis by up-regulating VEGF-C expression in human breast cancer. Thus, elevated MT1-MMP may serve as a significant prognostic factor in breast cancer.

## Background

Breast cancer invasion and metastasis is a complex process that begins with degradation of the basement membrane by multiple proteinases. The matrix metalloproteinases (MMPs) are one of the families of enzymes that mediate this process as they are capable of cleaving the extracellular matrix (ECM). Matrix 1 metalloproteinase (MT1-MMP) is particularly significant [1] as it directly degrades a number of ECM macromolecules, including collagen types I, II, III, laminins-1 and −5, fibronectin, vitronectin, fibrin and aggrecan [2,3]. MT1-MMP can also degrade the ECM indirectly by activating pro-MMP-2 on the cell surface [4]. MMP-2 activation is an important step for cancer invasion because this enzyme degrades type IV collagen, a major component of the basement membrane that is a barrier to invading cells [5]. In addition to degrading the ECM barrier to make a path, shedding of CD44 and syndecan-1 from the cell surface by MT1-MMP enhances cancer cell migration and invasion. Thus, accumulating evidence indicates that MT1-MMP is a critical factor for tumour invasion and metastasis [6].

Metastasis to regional lymph nodes via the lymphatic vessels is the predominant pattern of metastasis in breast cancer. Although the biochemical mechanisms are not well understood, the new lymphatic capillaries formed via lymphangiogenesis play a critical role in breast cancer lymphatic metastases. However, little is known about lymphangiogenesis due to the lack of markers with which to isolate and study lymphatic endothelium. Recently, this has been overcome after the identification of vascular endothelial growth factor C (VEGF-C) as a lymphangiogenic factor. VEGF-C is synthesised as propeptide, activated by proteolysis to form a high-affinity ligand that binds to the extracellular domain of vascular endothelial growth factor receptor 3 (VEGFR-3), which is predominantly expressed on lymphatic endothelia, and induces tyrosine phosphorylation of VEGFR-3. Thus, VEGF-C promotes intratumoural lymphangiogenesis and lymphatic metastasis in tumours [7].

Lymphangiogenesis is similar to angiogenesis in that it is a process of lymphatic endothelial cell (LEC) activation and proliferation, as well as the migration of newly formed capillaries through the physical barrier of the ECM to form the lymphatic vascular system. This process requires degradation of the interstitial matrix, which is the major role of MMPs such as MT1-MMP. We hypothesised that MT1-MMP also plays an important role in lymphangiogenesis through an interaction with VEGF-C. In this study, we investigated VEGF-C and MT-MMP levels in five breast carcinoma cell lines and correlated these levels with the invasive activity of breast cancer cells. Moreover, we examined the correlation between VEGF-C and MT1-MMP expression and compared these results with breast cancer tumour aggressiveness, clinicopathological features of breast cancer patients, and patient outcomes. Our findings suggest that VEGF-C and MT1-MMP expression is closely correlated and affects the prognosis of breast carcinomas, providing new insight into their effects during invasion and metastasis of breast cancer.

## Results

### Correlation between MT1-MMP and VEGF-C in breast cancer cell lines

**Figure 1:**
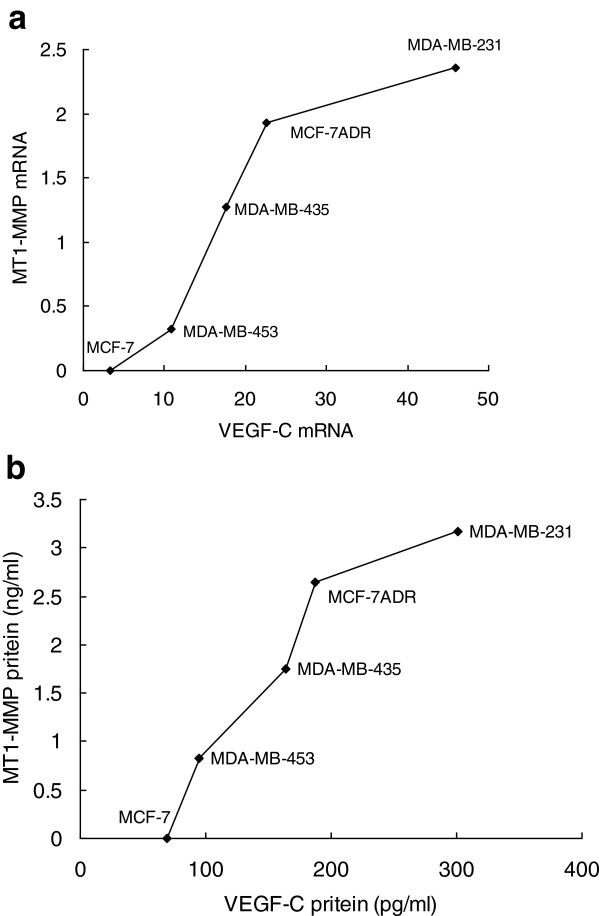
**The correlation between MT1-MMP and VEGF-C expression in human breast cancer cells. (a)** MT1-MMP and VEGF-C mRNA expression. **(b)** MT1-MMP and VEGF-C protein expression.

We used real-time RT-PCR to determine whether VEGF-C or MT1-MMP mRNA expression levels differed markedly among five breast cancer cell lines and correlated mRNA expression changes with protein levels measured by enzyme-linked immunosorbent assay (ELISA). MDA-MB-231 and MCF-7ADR cell lines had higher expression levels of VEGF-C and MT1-MMP, while neither MT1-MMP mRNA nor protein expression was detected in MCF-7 cells. VEGF-C mRNA expression correlated well with MT1-MMP mRNA expression (r = 0.914, P = 0.03; Figure 1a), and a similar correlation was found between the protein expression of VEGF-C and MT1-MMP (r = 0.945, P = 0.02; Figure 1b).

### Effects of MT1-MMP and VEGF-C on invasive phenotype of tumour cells

We found a statistically significant correlation between MT1-MMP mRNA expression in five breast cancer cell lines and the number of invasive tumour cells (r = 0.984. P = 0.002). VEGF-C mRNA expression was also closely associated with the number of invasive tumour cells (r = 0.979, P = 0.004).

The invasive activity of MDA-MB-231 cells into reconstituted basement membrane components was inhibited by 0, 4, 8, or 12 μg/ml anti-MT1-MMP antibody in a concentration-dependent manner. Moreover, RT-PCR showed that treatment of MDA-MB-231 with an anti-MT1-MMP antibody resulted in a concentration-dependent decrease in VEGF-C mRNA expression levels. An anti-VEGF-C antibody also inhibited the invasive activity of MDA-MB-231 cells, but did not affect MT1-MMP mRNA expression levels.

### Immunohistochemistry for MT1-MMP and VEGF-C

We assessed MT1-MMP and VEGF-C expression in samples from breast cancer patients. In normal mammary epithelial cells, we did not observe immunostaining for MT1-MMP and VEGF-C. However, diffuse cytoplasmic staining for VEGF-C protein was seen in tumour cells, while distinct tumour cell membrane staining of MT1-MMP was observed. We defined positive staining as immunoreactivity in >10% of the carcinoma cells [8]. Of the 106 breast cancer specimens, 55 (58.1%) had positive staining for VEGF-C and 66 (62.3%) for MT1-MMP. A significant correlation was found between the expression of VEGF-C and MT1-MMP (r = 0.458, P < 0.001).

### Correlation between MT1-MMP, VEGF-C expression and clinicopathological features

We correlated the expression of MT1-MMP and VEGF-C with clinicopathological features of breast cancer patients (Table 1). Immunoreactivity of MT1-MMP was strongly correlated with clinical stage and axillary lymph node metastasis. VEGF-C expression was also strongly correlated with clinical stage, lymph node metastasis and tumour size. There was also a close association between VEGF-C, MT1-MMP expression and lymphatic vessel density.

**Table 1:** Correlations between VEGF-C and MT1-MMP expression and the clinicopathological features of 106 breast cancers

	**VEGF-C expression**			**MT1-MMP expression**		
	**Positive**	**Negative**	***P***	**Positive**	**Negative**	***P***
Tumour						
T1	14 (60.9%)	9 (39.1%)	0.023	11 (47.8%)	12 (52.2%)	0.247
T2	30 (51.7%)	28 (48.3%)		23 (39.7%)	35 (60.3%)	
T3	7 (41.2%)	10 (58.8%)		5 (29.4%)	12 (70.6%)	
T4		8 (100%)		1 (12.5%)	7 (87.5%)	
N						
N0	25 (67.6%)	12 (32.4%)	0.002	23 (62.2%)	14 (37.8%)	<0.001
N1	21 (45.7%)	25 (54.3%)		15 (32.6%)	31 (67.4%)	
N2	5 (21.7%)	18 (78.3%)		2 (8.7%)	21 (91.3%)	
Stage						
I	7 (77.8%)	2 (22.2%)	0.002	6 (66.7%)	3 (33.3%)	<0.001
II	39 (54.2%)	33 (45.8%)		32 (44.4%)	40 (55.6%)	
III	5 (20.0%)	20 (80.0%)		2 (8.0%)	23 (92.0%)	
ER						
Positive	21 (35.0%)	39 (65.0%)	0.002	41 (68.3%)	19 (31.7%)	0.1409
Negative	30 (65.2%)	16 (34.8%)		25 (54.3%)	21 (45.7%)	
PR						
Positive	27 (42.2%)	37 (57.8%)	0.132	43 (67.2%)	21 (32.8%)	0.1967
Negative	24 (57.1%)	18 (42.9%)		23 (54.8%)	19 (45.2%)	
Her2						
Positive	24 (42.1%)	33 (57.9%)	0.181	37 (64.9%)	20 (35.1%)	0.5440
Negative	27 (55.1%)	22 (57.9%)		29 (59.2%)	20 (40.8%)	

### Correlation between MT1-MMP, VEGF-C expression and patient outcome

Of the 106 patients examined in this study, 42 (39.6%) died due to relapse of the disease during the follow-up period. The overall survival rates for the 66 patients with positive MT1-MMP tumour staining were significantly lower than that of 40 patients with negative MT1-MMP staining (P = 0.0002), while the prognosis of 55 patients with positive VEGF-C tumour staining was poorer than with negative VEGF-C staining (P = 0.0001). Patients with tumours simultaneously positive for MT1-MMP and VEGF-C had the lowest overall survival rates. In contrast, patients with negative MT1-MMP and VEGF-C tumour expression had the highest survival rates (P = 0.0001). There was no difference in the clinical outcome between patients with positive MT1-MMP and negative VEGF-C tumour expression or patients with negative MT1-MMP and positive VEGF-C tumour expression.

## Discussion

Tumour invasion and metastasis are critical steps in determining the aggressive phenotype of human cancers and are the major causes of cancer deaths. It is well established that MT1-MMP can increase breast cancer cell migration, invasion and metastasis through several mechanisms including dissolving the basement membrane [9], cleavage of ECM components such as laminin 5 or type IV collagen [10,11], facilitating multicellular strand formation [12], and processing of cell adhesion molecules such as CD44 [13,14] and integrin subunits [15,16]. In our study, a positive correlation between MT1-MMP protein expression in five breast cancer cell lines and the number of invasive tumour cells showed that higher expression levels of MT1-MMP were associated with increased cell invasion, consistent with previous reports [17-19].

Promotion of tumour metastasis by VEGF-C is a consequence of tumour lymphangiogenesis via activated VEGFR-3, which is located on lymphatic endothelial cells [20]. In the present study, we showed that higher expression levels of VEGF-C correlated with increased cell invasion in breast cancer cell lines and that antibody inhibition of VEGF-C resulted in reduced tumour cell invasive activity. These results demonstrated that VEGF-C promotes breast cancer metastasis by inducing lymphangiogenesis, as well as enhancing cancer cell mobility and invasiveness. Greater baseline invasion of cancer cell lines that express high levels of VEGF-C is consistent with published reports [21] in lung cancer cells lines, breast cancer cell lines [22] and head and neck cancer cell lines [23]. Recent studies show that VEGFR-3 is expressed on tumour cells in a variety of human malignancies, including non-small cell lung cancer tumours [24,25], breast cancer [26], colorectal adenocarcinoma [27,28], head and neck carcinomas [29,30], and prostate carcinoma [31]. These studies indicate that tumour cell expression of VEGFR-3 supports VEGF-C autocrine signalling, which promotes tumour cell invasion and motility in cancer. The mechanisms responsible for this may include activation of the p38 mitogen-activated protein kinase (MAPK) pathway by the VEGF-C/Flt-4 axis [21], which mediates cell migration and invasion in various cancer cells [32,33].

Although many studies of the roles of MT1-MMP and VEGF-C in malignancies have been reported, the clinicopathological significance of MT1-MMP and VEGF-C in human tumours is still a subject of debate. In breast cancer, several studies clearly demonstrate a significant association between high MT1-MMP expression, positive lymph node status, and a poor prognosis for disease-free survival (DFS) and overall survival (OS) [34-38]. Other studies report a negative relationship between MT1-MMP expression and lymph node involvement or its use as a useful prognosticator in breast cancer [35-37,39,40]. The majority of studies have demonstrated a significant association between VEGF-C expression and lymph node metastasis or a poor prognosis in breast cancer [41-49], while others failed to relate VEGF-C expression to positive lymph node status and a poor prognosis in [50-53]. This discrepancy may lie in the failure to use standardised methods of collecting and analysing data, as well as the use of different antibodies.

In our study, MT1-MMP and VEGF-C overexpression were both significantly associated with lymph node metastasis and a poor prognosis for DFS and OS. Those patients with negative MT1-MMP and VEGF-C had the largest DFS and OS rates. Little is known about the mechanism of metastasis via the lymphatic vessels. The simple explanation is that lymphangiogenesis in breast cancer increases the contact area between invading tumour cells and the lymphatic endothelium. In addition, lymphatic endothelial cells might attract tumour cells by secreting chemokines that actively promote lymphatic metastasis [54,55]. In our study, elevated MT1-MMP expression was positively associated with LVD, suggesting that MT1-MMP promotes the formation of lymphatic vessels.

During lymphatic vessel formation, LECs send long filopodia towards the VEGF-C-producing tumour tissues and form tumour-directed vascular sprouts [56]. We presume that the process of lymphangiogenesis must consist of the following steps: (i) LEC proliferation, (ii) LEC migration and ECM degradation [57], (iii) LEC sprouting and invading into ECM, (iv) capillary lumen formation. Thus, degradation of matrix proteins is a critical step in lymphangiogenesis. Many specific proteolytic enzymes are involved in this degradation process. MMPs are a large family of proteolytic enzymes that play a key role in the degradation of ECM [57]. New data show that the MT1-MMP is a major modifier of the pericellular environment. It is well established that degradation of the basement membranes is an essential requirement for the formation of new vessels and MT1-MMP is a key mediator of matrix degradation during the angiogenic response [58], suggesting that MT1-MMP plays a role in lymphangiogenesis in human breast cancer [59]. Our study further shows that MT1-MMP expression was well correlated with VEGF-C expression in breast cancer cells, and significantly correlated in breast tumours. Our results are consistent with a report that elevated MT1-MMP expression was associated with elevated VEGF-C in angiogenesis [58]. Furthermore, we found that inhibition of MT1-MMP expression in breast cancer cell lines resulted in a decrease in VEGF-C expression in a concentration-dependent manner. These findings imply that MT1-MMP affects lymphangiogenesis by regulating VEGF-C expression. Therefore, MT1-MMP is an important factor involved in lymphangiogenesis and results in higher lymph vessel density (LVD).

## Conclusions

In conclusion, our study reveals that MT1-MMP and VEGF-C enhance the invasive potential of breast cancer cells *in vitro* and have prognostic value for breast cancer patients. Both MT1-MMP and VEGF-C were positively associated with LVD and lymph node metastasis, and MT1-MMP may promote lymphangiogenesis by up-regulating VEGF-C expression in human breast cancer. Further studies should investigate the mechanisms underlying VEGF-C protein processing by MT1-MMP in human cancer.

## Materials and methods

### Cell culture

Human breast adenocarcinoma cells lines (MCF-7, MDA-MB-453, MDA-MB-435, MCF-7ADR, MDA-MB-231) were stored in our laboratory. All cells were cultured in Dulbecco’s modified Eagle’s medium (DMEM) supplemented with penicillin, streptomycin, 50 ng/ml ascorbic acid and 10% foetal calf serum (FCS) (Gibco BRL, Grand Island, NY, USA). Cells were maintained in a humidified incubator at 37°C with 5% CO_2_. The absence of mycoplasma was confirmed using the Genprobe kit (Gen-Probe, San Diego, CA, USA).

### Real-time RT-PCR assessment of MT1-MMP and VEGF mRNA expression

The expression of MT1-MMP and VEGF-C transcripts was determined using real-time quantitative PCR. Briefly, total RNA was extracted using TRIzol reagent (Invitrogen, Grand Island, NY, USA) according to the manufacturer’s instructions. Total RNA (1 μg) was reverse-transcribed into single-stranded cDNA with oligo-dT_18_ primer and SuperScript II reverse transcriptase (Invitrogen). Amplification of MT1-MMP, VEGF-C and glyceraldehyde-3-phosphate dehydrogenase (GAPDH) as an internal control in each reaction was carried out by polymerase chain reaction (PCR) with the following primers: MT1-MMP, 5′-CCTGCATCCATCAATACTACTGC-3′ (forward) and 5′-GCGTCTGAAGAAGAAGAC AGC-3′ (reverse); VEGF-C 5′-CAGTTACGGTCTGTGTCCAGTGTAG- 3′ (forward) and 5′-GGACACACATGGAGGTTTAAAGAAG-3′ (reverse); GAPDH 5′-CCACCCATGGCAAATTCCATGGCA-3′ (forward), 5′-TCTAGACGGCAGGTCAGGTCCACC-3′ (reverse). Primers were used at a final concentration of 0.5 μM. The reaction mixture was first denatured at 95°C for 10 min followed by amplification at 95°C for 1 min, 55°C for 1 min, and 72°C for 1 min for 30 cycles, and by 72°C for 10 min. PCR products were visualised on a 2% agarose gel containing 1 μg/mL ethidium bromide. To evaluate the mRNA expression levels of MT1-MMP and VEGF-C, the ratios of MT1-MMP and VEGF-C GAPDH mRNA expression were measured by real-time quantitative PCR using a Light Cycler system and reagents (Roche Molecular Diagnostics) with the double-stranded DNA binding dye, SYBR Green 1, according to the procedure provided by the manufacturer. Standard curves were prepared for both the target gene (MT1-MMP and VEGF-C) and the internal control (GAPDH) by amplifying four logarithmic dilutions of plasmid containing the target fragment as templates. Standard dilutions were optimised to cover the relevant concentration range of target and reference RNA in the cell. The quantities of MT1-MMP and VEGF-C were determined from the standard curve and divided by the quantity of the GAPDH internal control. The quantities of MT1-MMP and VEGF-C were expressed as a fold differences relative to GAPDH from three experiments in duplicate.

### MT1-MMP activity assay

Cells were plated onto 15-cm plastic dishes and grown to confluence. Plasma membrane preparations were collected with a cell scraper, and suspended in 1-ml ice-cold phosphate-buffered saline (PBS) containing protease inhibitors. The lysates were further sonicated for 5 s, and the membranes pelleted by centrifugation at 14,000 rpm for 30 min at 4°C. Pelleted membranes were washed once and resuspended in 0.5-ml complete PBS. Protein concentrations were determined with a bicinchoninic acid reagent, and samples were diluted with complete PBS to obtain starting concentrations of 1 mg/ml. The MT-MMP activity assay was performed according to the manufacturer’s protocol (Chemicon, Massachusetts, USA). Briefly, varying dilutions of the starting membrane preparations were APMA-activated and incubated for 1 h at 37°C with the MT-MMP peptide substrate (MCA-Pro-Leu-Gly-Leu-Dpa-Ala-Arg-NH2) in a 96-well plate, and read the plate on a fluorometric plate reader using a 325 nm excitation and 395 nm emission filters. Standard curves with the control peptide and MT1-MMP positive control were generated to determine the appropriate sample dilution at which the values fell in the middle of the standard curves. The experiments were completed in triplicate and repeated once.

### ELISA for VEGF-C

Concentrations of VEGF-C in the medium of the cultured tumour cells were determined using a VEGF-C ELISA kit, as described in the protocol provided by the manufacturer (Bender MedSystems, San Diego, CA, USA). Briefly, cell culture medium was diluted 50-fold with sample diluent and added into wells of a microwell plate coated with anti-VEGF-C polyclonal antibody and incubated for 2 h at room temperature. Biotin-conjugated polyclonal VEGF-C antibody was added and the plate incubated for 1 h at room temperature. The plate was washed and streptavidin-HRP added. Following incubation, unbound streptavidin-HRP was removed during a wash step, and the colour reagent containing HRP added to the wells. The reaction was terminated by addition of stop solution and absorbance is measured at 450 nm in an Elx808IU Ultra Microplate Reader (BIO-TEK Instruments, Inc., Winooski, Vermont, USA). A standard curve was constructed using the VEGF-C protein standard provided in the kit. Generally, the samples were analysed using different dilutions in triplicate.

### Membrane invasion culture system assay

The membrane invasion culture system chamber was used to measure *in vitro* invasiveness of the breast cancer cells. Briefly, transwell inserts (Corning, Union City, CA, USA) with 8 μm pores were uniformly coated with 50 μg of Matrigel (Becton Dickinson, San Jose, CA, USA) and air-dried before being rehydrated. Single-tumour-cell suspensions, prepared after trypsinisation, were seeded into the upper wells at a concentration of 1 × 10^5^ per well. After a 24-h incubation in a humidified incubator at 37°C with 5% CO_2_, the inserts were fixed in methanol and stained for 5 min with eosin followed by 5 min with haematoxylin. The cells on the upper surface of the inserts were wiped away with a cotton swab. The cells that had migrated through the matrix and adhered to the lower surface of the inserts were counted as nine separate fields at 40× magnification. Each cell line was tested at least twice and within a single experiment, each assay was performed in quadruplicate.

### Effects of anti–VEGF-C and anti–MT1-MMP on the invasive phenotype of breast cancer cells

The effects of an anti-MT1-MMP antibody (Chemicon, Massachusetts, USA) and anti-VEGF-C antibody (Santa Cruz Biotechnology, Dallas, Texas, USA) on the invasive and proteolytic activity of tumour cells were examined using the membrane invasion assay. Briefly, log-phase cell cultures of MDA-MB-231 were harvested. A 200-μL cell suspension was added to transwell inserts and incubated with anti-MT1-MMP antibody at concentrations of 0, 4, 8, or 12 μg/ml for 24 h. Invasive cells were counted as described above. VEGF-C mRNA levels in cells incubated with various amounts of anti-MT1-MMP antibody were analysed by real-time RT-PCR as described above. Similarly, the effects of anti-VEGF-C antibody on invasive activity and MT1-MMP mRNA expression were also examined.

### Patients and tissue samples

Formalin-fixed, paraffin-embedded samples obtained from 106 invasive ductal carcinomas and normal tissues from benign breast disease procedures free from pathological changes (n = 43) were retrieved from the pathology files of Nanfang Hospital, Southern Medical University (Guangzhou, China). All breast cancer patients underwent mastectomy with an axillary dissection during 2004 and none received pre-operative radiation or chemotherapy. The patients with histologically positive axillary lymph nodes or a primary tumour > 1 cm in diameter received post-operative chemotherapy. All patients were treated with adjuvant anti-oestrogen therapy if oestrogen receptor or progesterone receptor. All women were followed-up after surgical treatment at 6-month intervals for a mean period of 71.41 months. All patients provided informed consent according to a protocol approved by the ethics committee of the institute. Details of breast cancer patients are provided in Table 2.

**Table 2:** Patients’ characteristics

**Parameter**	**Number of patients (%)**
Sex	
Female	106(100%)
Histologic subtype	
IDC	106(100%)
Median age	47.1(25–81)
Menopausal status	
Pre	43(40.6%)
Post	63(59.4%)
Stage	
I	9(8.5%)
II	72(67.9%)
III	25(23.6%)
Estrogen receptor status	
Positive	60(56.6%)
Negative	46(43.4%)
Progesterone receptor status	
Positive	64(60.4%)
Negative	42(39.6%)
Her2	
Positive	57(53.8%)
Negative	49(46.2%)

IDC = infiltrating ductal carcinoma.

### Immunohistochemical staining for VEGF-C, MT1-MMP and D-20

To identify the expression of MT1-MMP and VEGF-C in the breast cancer tissue specimens, immunohistochemical analysis was performed. Briefly, paraffin-embedded breast cancer samples were cut into 4-μm sections. Slides were incubated for 12 h at 37°C, de-waxed in a histological clearing agent and hydrated. Endogenous peroxidase activity was blocked by incubation in 3% hydrogen peroxide for 15 min. Non-specific binding was prevented by incubation in 1.5% normal rabbit serum (VEGF-C) or 3% normal mouse serum (MT1-MMP) for 15 min in a humidified chamber in PBS. Slides were incubated with anti-VEGF-C (1.14 μg/ml; Dallas, Texas, USA) or MT1-MMP (5 μg/ml; Oncogene, Cambridge, MA, USA) for 60 min at room temperature. The primary antibody was detected using a biotinylated horse anti-rabbit (VEGF-C) or horse anti-mouse (MT1-MMP) secondary antibody for 30 min at room temperature and the peroxidase was introduced using a streptavidin conjugate. The slides were washed thoroughly with PBS between each stage in the procedure. The antibody reaction was visualised using a fresh substrate solution containing an aminoethyl carbazole substrate kit (Sigma, St. Louis, MO, USA). The sections were counterstained with haematoxylin, dehydrated and mounted in glycerol–vinyl–alcohol. For the negative controls, the primary antibody was replaced with mouse IgG.

A monoclonal mouse anti-human D2-40 antibody (Zymed, Grand Island, NY, USA) was used for the staining of lymphatic vessels. The D2-40 antibody detects a fixation-resistant epitope on a 40 kDa O-linked sialoglycoprotein expressed in lymphatic endothelium but not blood vessels, and can be used to assess lymphangiogenesis specifically in conventionally processed formalin-fixed and paraffin-embedded tissue specimens [60,61]. The procedure for immunohistochemical staining of D2-40 is similar to that for MT1-MMP and VEGF-C. Sections from a previously studied case of tonsilla known to express D2-40 were used as positive controls.

Two pathologists who were unaware of the clinical data evaluated the immunohistochemical staining. To evaluate MT1-MMP and VEGF-C protein expression, the results were graded as follows; (+), >10% of the neoplastic cells were stained; (±), <10% of the neoplastic cells were stained; (−), neoplastic cells were not completely stained. In this study, (−) and (±) were classified as negative.

Lymphatic vessel density (LVD) was determined as suggested by Weidner *et al.*[62]. The immunostained sections were scanned by light-microscopy at low magnification (40×) and the areas of tissue with the greatest number of distinctly highlighted microvessels (‘hot spots’) were selected. LVD was then determined by counting all immunostained vessels at a total magnification of (200×) from five areas for each case.

### Statistical analysis

All statistical calculations were carried out using the SPSS statistical software. The relation between two different values was evaluated using the Pearson correlation coefficient. Correlations between VEGF-C, MT1-MMP expression and clinicopathological features were assessed by the Chi-square test. Survival curves were generated by the Kaplan-Meier method and the difference between the curves was assessed using the log-rank test. P < 0.05 was accepted as indicative of statistical significance.

### Ethical approval

Department of Scientific Research approved the study and the publication of our paper.

## Abbreviations

ECM: Extracellular matrix; LEC: Lymphatic endothelial cell; LVD: Lymph vessel density; MMP: Matrix metalloproteinases; MT1-MMP: Membrane-type matrix 1 metalloproteinase; VEGF-C: Vascular endothelial growth factor C.

## Competing interests

The authors declare that they have no competing interests.

## Authors’ contributions

CY and GY conceived and designed the study. GY and PH drafted the manuscript. GY, PH, LC, XH, FG performed the experimental studies. All authors have read and approved the final manuscript.
